# Practical Liposomal Formulation for Taxanes with Polyethoxylated Castor Oil and Ethanol with Complete Encapsulation Efficiency and High Loading Efficiency

**DOI:** 10.3390/nano7100290

**Published:** 2017-09-23

**Authors:** Tsukasa Shigehiro, Junko Masuda, Shoki Saito, Apriliana C. Khayrani, Kazumasa Jinno, Akimasa Seno, Arun Vaidyanath, Akifumi Mizutani, Tomonari Kasai, Hiroshi Murakami, Ayano Satoh, Tetsuya Ito, Hiroki Hamada, Yuhki Seno, Tadakatsu Mandai, Masaharu Seno

**Affiliations:** 1 Department of Medical Bioengineering, Graduate School of Natural Science and Technology, Okayama University, Okayama 700-0082, Japan; en20727@s.okayama-u.ac.jp (T.S.); junkomasuda@okayama-u.ac.jp (J.M.); p4hi0alp@s.okayama-u.ac.jp (S.S.); apriliana41@gmail.com (A.C.K.); ptix7zkp@s.okayama-u.ac.jp (K.J.); aseno@okayama-u.ac.jp (A.S.); arunvnath@okayama-u.ac.jp (A.V.); mizut-a@cc.okayama-u.ac.jp (A.M.); t-kasai@cc.okayama-u.ac.jp (T.K.); muraka-h@cc.okayama-u.ac.jp (H.M.); ayano113@cc.okayama-u.ac.jp (A.S.); 2 Japan Society for the Promotion of Science, Tokyo 102-0083, Japan; 3 Ensuiko Sugar Refining Co., Ltd., Tokyo 102-0083, Japan; tetsuya_ito@pearlace.co.jp; 4 Faculty of Science, Okayama University of Science, Okayama 700-0082, Japan; hamada@dls.ous.ac.jp; 5 Faculty of Life Science, Kurashiki University of Science and the Arts, Kurashiki 712-8505, Japan; s20b028@sci.kusa.ac.jp (Y.S.); ted@chem.kusa.ac.jp (T.M.)

**Keywords:** liposomal drug delivery, targeted drug delivery, taxanes, Cremophor EL, HER2

## Abstract

Taxanes including paclitaxel and docetaxel are effective anticancer agents preferably sufficient for liposomal drug delivery. However, the encapsulation of these drugs with effective amounts into conventional liposomes is difficult due to their high hydrophobicity. Therefore, an effective encapsulation strategy for liposomal taxanes has been eagerly anticipated. In this study, the mixture of polyethoxylated castor oil (Cremophor EL) and ethanol containing phosphate buffered saline termed as CEP was employed as a solvent of the inner hydrophilic core of liposomes where taxanes should be incorporated. Docetaxel-, paclitaxel-, or 7-oxacetylglycosylated paclitaxel-encapsulating liposomes were successfully prepared with almost 100% of encapsulation efficiency and 29.9, 15.4, or 29.1 mol% of loading efficiency, respectively. We then applied the docetaxel-encapsulating liposomes for targeted drug delivery. Docetaxel-encapsulating liposomes were successfully developed HER2-targeted drug delivery by coupling HER2-specific binding peptide on liposome surface. The HER2-targeting liposomes exhibited HER2-specific internalization and enhanced anticancer activity in vitro. Therefore, we propose the sophisticated preparation of liposomal taxanes using CEP as a promising formulation for effective cancer therapies.

## 1. Introduction

The liposomal drug delivery system has a great potential for cancer chemotherapy [1,2]. Encapsulation of the chemotherapeutic drugs into liposomes can alleviate side effects because the drug release from liposomes is controlled [1]. Further, liposomes whose particle size between 100 and 200 nm are passively accumulated into tumor tissues due to enhanced permeability and retention (EPR) effects [3]. However, the conventional liposomes are rapidly eliminated from blood by the reticuloendothelial system (RES) including kupffer cells in the liver [4]. Polyethylene glycol (PEG) coated liposomes known as long-circulating liposomes are often applied for liposomal drug delivery system, which can escape RES uptake by generating steric barrier and water-phase on liposome surface [5].

PEGylated liposomal doxorubicin is the first approved liposomal drug formulation for cancer therapy to effectively encapsulate the drug into liposomes with an active loading method under ammonium sulfate gradient or pH-gradient between the inner hydrophilic core and the outer solvent of the liposomes [6,7]. The encapsulation strategy has been developed for other weak base drugs including daunorubicin and vincristine, which are also approved as liposomal formulations for cancer therapy [6,8]. Furthermore, the liposomal doxorubicin has been developed as functional liposomal formulations to specifically deliver to the target tumor and release the encapsulated drug in the tumor [9,10]. Doxorubicin encapsulating PEGylated liposomes conjugated to GAH, which is a cancer-specific human monoclonal antibody, has been evaluated under clinical trial [11]. Therefore, the liposomal formulation is now considered as an important part of drug development under encapsulation strategy [12,13,14,15]. 

Taxanes including paclitaxel (PTX) and docetaxel (DTX) exhibit high cytotoxicity by inhibiting tubulin depolymerization [16,17]. Liposomes encapsulating taxanes are considered as a promising application of nanoparticles for cancer therapy [18]. Notwithstanding extensive investigation of the formulation of liposomal taxanes, encapsulation of taxanes into liposomes is quite difficult due to the high hydrophobicity without basic or acidic character. The liposomal formulation of hydrophobic compounds such as taxanes has recently been devised to incorporate the drugs into the lipid bilayer of liposomes [19]. However, the amount of taxanes incorporated into the liposomes is substantially limited because the more the drugs are incorporated, the more the lipid membrane becomes unstable. Actually, this method was not applicable to generate PEGylated liposomes encapsulating PTX [20]. Therefore, it is impossible for PEGylated liposomes containing taxanes to develop targeted drug delivery with conventional liposomal formulation because of instability of the membrane.

Incorporation of surfactants, lysolipids, and phospholipids with short chain fatty acid into lipid bilayer improves the drug capacity of liposomes for taxanes because the transition temperature (Tm) of membrane gets down to increase the fluidity of the lipid bilayer [21,22,23]. For example, a medium chain triglyceride, Captex 300 (C300), strongly enhanced the drug capacity of both PTX and DTX when incorporated into the liposome bilayer at the ratio of C300 to lipid 1 mg to 40 μmol, where the lipid was composed of 1,2-dimyristoyl-sn-glycero-3-phosphocholine (DMPC) and cholesterol (Chol) at the ratio of 7:1 mol% [23]. The incorporation of C300 should decrease the Tm of the lipid bilayer to give fluidity of the lipids allowing to accommodate PTX and DTX in the membrane. Encapsulation of surfactant into the hydrophilic core of liposomes has also been applied to generate liposomal taxanes. Tween 80, a surfactant, has been exploited as a solvent to suspend lipid film containing PTX [24]. Since 3 volume% of Tween 80 enhanced solubility of PTX, this method successfully encapsulated PTX into PEGylated liposomes. Further, conjugated with the anti-HER2 antibody, trastuzumab on the surface, the liposomes encapsulating PTX were devised as HER2-targeted drug delivery [25,26]. However, their HER2-targeting liposome encapsulating PTX was demonstrated in vivo experiment in mice at the dose of 7.5 mg/kg at single injection from tail vain, which is lower than the maximal tolerated dose of PTX in Cremophor EL based administration (20 to 35 mg/kg at single injection) [27,28]. The encapsulated amounts of the drug should be more to choose sufficient dose of administration for in vivo experiments even with the different solvent. Likewise, DTX, which is considered more hydrophilic than PTX, has been found insufficient to prepare the liposomes with good amounts of drug encapsulated [29,30,31,32]. Therefore, an effective encapsulation strategy has been sought to develop a suitable liposomal formulation of taxanes for drug delivery.

We previously reported that polyethoxylated castor oil (Cremophor EL) and ethanol containing phosphate buffered saline (PBS) termed as CEP (Cremophor EL:Ethanol:PBS = 20:15:65 volume%) exhibited great potential as a solvent to generate mono-glycosylated taxanes encapsulating liposomes [13,33]. The glycosylated PTX or DTX were encapsulated into liposomes in good efficiency by the active loading method under solubility gradient between CEP in the inner hydrophilic core and either 40% or 35 % ethylene glycol containing PBS in the outer membrane of the liposomes. Liposomes encapsulating glycosylated PTX or DTX prepared through the method allowed us to demonstrate experiments in vivo for the evaluation of antitumor activity with high dose administration of the drugs (75 mg/kg at single injection from tail vein). However, this liposomal drug formulation was not applicable for original PTX and DTX. Given successful development of PEGylated liposomes containing PTX using Tween 80 and of gPTX encapsulating liposomes using CEP that significantly enhances solubility of PTX/DTX, in this study, we developed the method to prepare PEGylated liposomes encapsulating effective amounts of taxanes by suspended taxane containing lipid in CEP. Further, to evaluate the potential of the liposomal taxanes for development of targeted drug delivery by further modification on liposome membrane, the resulting DTX-encapsulating liposomes were then coupled with an HER2 specific peptide and evaluated for HER2 targeting in vitro.

## 2. Results

### 2.1. Liposomes Encapsulating Taxanes

**Figure 1:**
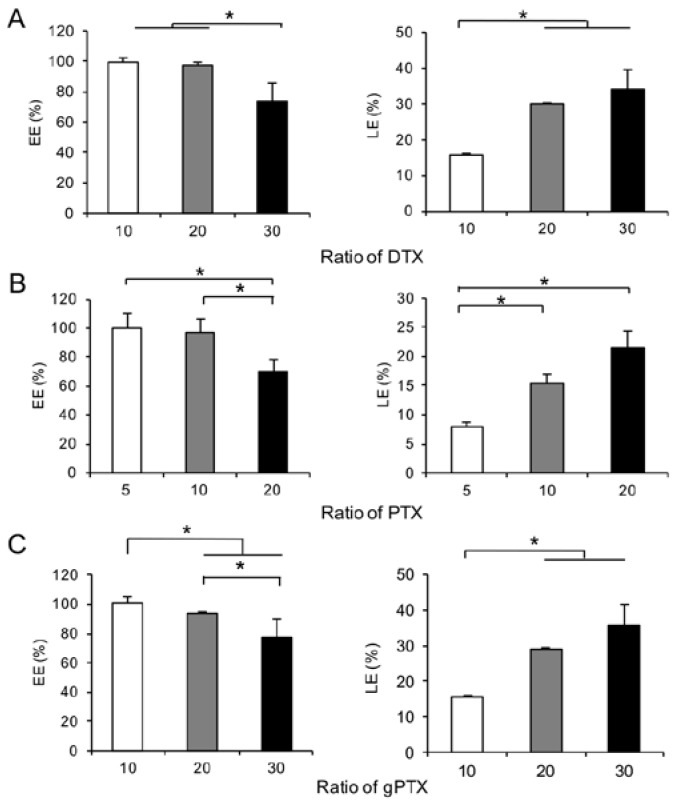
Encapsulation efficiency (EE) and loading efficiency (LE) of taxanes encapsulated into liposomes under different drug to lipid ratios. Liposomes composed of hydrogenated soybean phosphatidylcholine (HSPC), Chol, 1,2-distearoyl-*sn*-glycerol-3-phosphoethanolamine-*N*-[methoxy (polyethylene glycol)-2000] (mPEG-DSPE) and taxanes at the molar ratio of 60:40:5:*x*, *x* were prepared using CEP (20:15:65 volume%). (**A**) DTX-L; (**B**) PTX-L; (**C**) gPTX-L. Data are shown as mean ± S.D. where *N* = 4. * *p* < 0.05.

We attempted to prepare liposomes encapsulating taxanes as much as possible, using CEP containing clinically available surfactant, which is Cremophor EL, to suspended lipid film containing taxanes. The ratio of each component in CEP is the same as that in our previous study, which demonstrated the highest encapsulation efficiency of glycosylated DTX under the solubility gradient method [33]. As the results, DTX, PTX, and glycosylated PTX (gPTX) could be encapsulated into liposomes in good encapsulation efficiency (EE) and loading efficiency (LE) (Figure 1). To achieve over 90% of EE, the maximal drug to lipid ratio was found at 20, 10 and 20 for DTX, PTX and gPTX, respectively. The LEs of DTX, PTX, and gPTX at the respective composition ratio were 29.9, 15.4, 29.1 mol%. The particle size of the liposomes ranged between 100 and 200 nm with polydispersity index (PDI) less than 0.2 and the surface of the liposomes was negatively charged in all of the taxanes (Table S1). 

**Figure 2:**
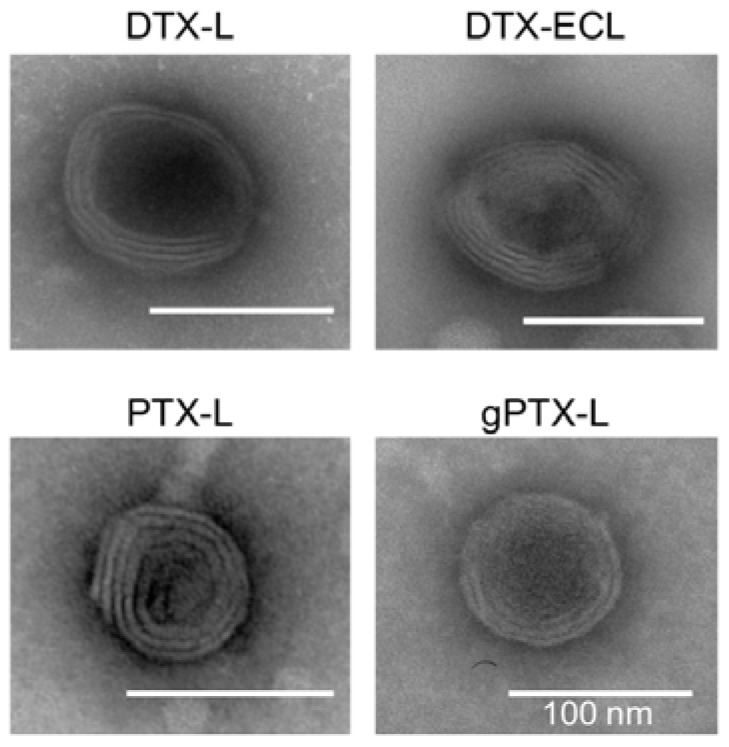
TEM images of liposomes encapsulating taxanes. Liposomal formulations of DTX-L, DTX-ECL, PTX-L, and gPTX-L were observed under TEM. All bars indicate 100 nm.

**Figure 3:**
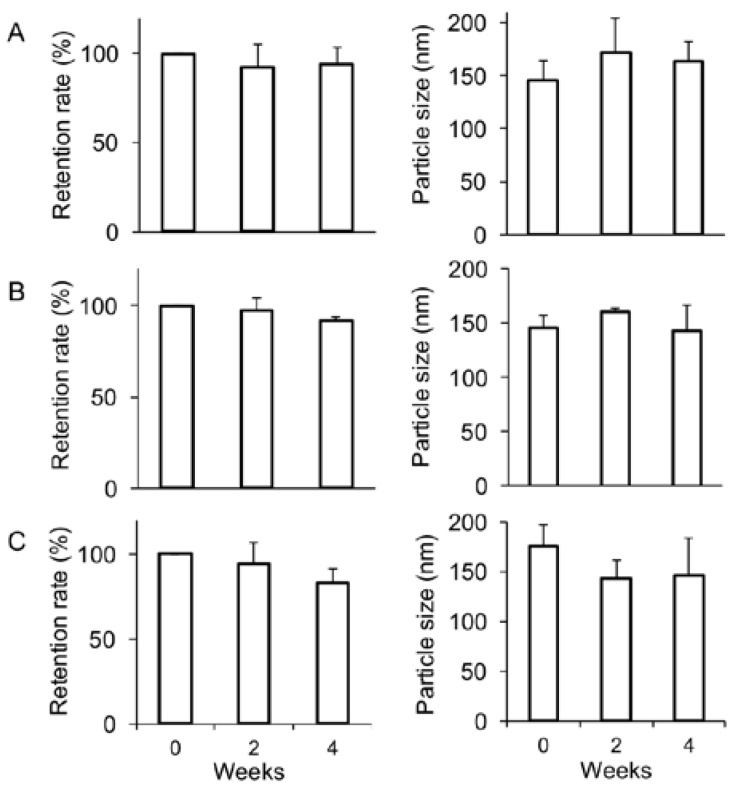
Stability of liposomes encapsulating taxanes at 4 °C in PBS. Stability of DTX-L (**A**) PTX-L. (B) and gPTX-L (**C**) was evaluated at 4 °C in PBS after 2- and 4-week incubation. The retained drug amounts in liposomes were estimated by reverse-phase HPLC. The distributions of particle sizes and particle number were determined by dynamic light scattering method. *N* = 3.

PEGylated liposomes encapsulating PTX have effectively been prepared with 3 volume% Tween 80 [24]. The formation of multi-lamellar vesicles (MLVs) was observed under microscope just after suspension of lipid film containing PTX or DTX with either CEP or 3 volume% Tween 80 (Figure S1). While needle-like aggregations, which might be precipitations of lipids/taxane, were majorly found when suspended in 3 volume% Tween 80, MLVs were clearly observed when suspended in CEP in our lipid/drug composition ratio. After preparation of the liposomes encapsulating taxanes with CEP, morphology of the liposomes was observed under a transmission electron microscopy (TEM) (Figure 2). These liposomal taxanes exhibited cytotoxicity equivalent to or higher than naked drugs when evaluated by the MTT assay (Figure S2). Furthermore, any precipitates of taxanes were not visually observed after 2- and 4-week incubation at 4 °C. Even under TEM image of PTX-L, any aggregates/precipitates of the drug in liposomes were not found about 2 to 3 weeks after preparation (data not shown). Confirmed by reverse-phase HPLC, the encapsulated taxanes in liposomes were found stably maintained without significant leakage when kept at 4 °C for 4 weeks (Figure 3). 

We then tried to apply DTX-L for the formulation targeting HER2 exploiting EC1 peptide with specific affinity to HER2 [34]. EC1 peptide fused to human IgG Fc domain (EC-Fc) was conjugated on the surface of DTX-L to design EC1 coupling DTX-L (DTX-ECL) for HER2-targeted drug delivery. The characteristics of DTX-ECL did not significantly change when compared with those of DTX-L sustaining high EE at approximately 70% (Figure 2 and Table 1).

### 2.2. Cytotoxicity of the Formulations for DTX

**Figure 4:**
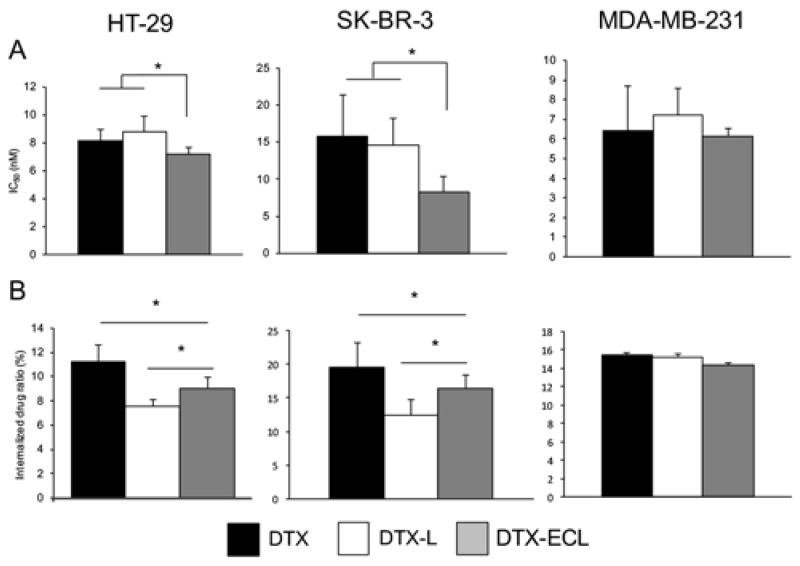
Cytotoxicity and internalized drug amount in HER2 positive cancer cells (HT-29 and SK-BR-3) and HER2 negative cancer cells (MDA-MB-231) treated with DTX-ECL. The cytotoxicity of DTX-ECL after 3-h drug exposure was evaluated by the MTT assay (**A**); The internalized drug amounts after 3-h drug exposure were determined by reverse-phase HPLC (**B**). * *p <* 0.05; *N* = 4.

The cytotoxicity of DTX, DTX-L, and DTX-ECL was assessed on HER2 positive cancer cell lines, HT-29 cells and SK-BR-3 cells, and HER2 negative cancer cell line, MDA-MB-231 cells (Figure 4A) [35,36]. DTX-ECL exhibited significantly lower IC_50_ than DTX and DTX-L in HT-29 cells and SK-BR-3 cells. On the other hand, all of the drug formulations exhibited equivalent IC_50_ in MDA-MB-231 cells. To evaluate specific internalization of DTX-ECL to HER2 positive cancer cells, the uptake of the drug amount in the cells were evaluated after 3 h of drug exposure (Figure 4B). DTX exhibited the uptake at the most amount of the drug in all of the drug formulations. DTX-ECL exhibited the uptake of drug amount more than DTX-L in HT-29 cells and SK-BR-3 cells. The amounts of DTX in all of the drug formulations were almost equivalent in MDA-MB-231 cells.

### 2.3. Internalization of Liposomes into the Cells

**Figure 5:**
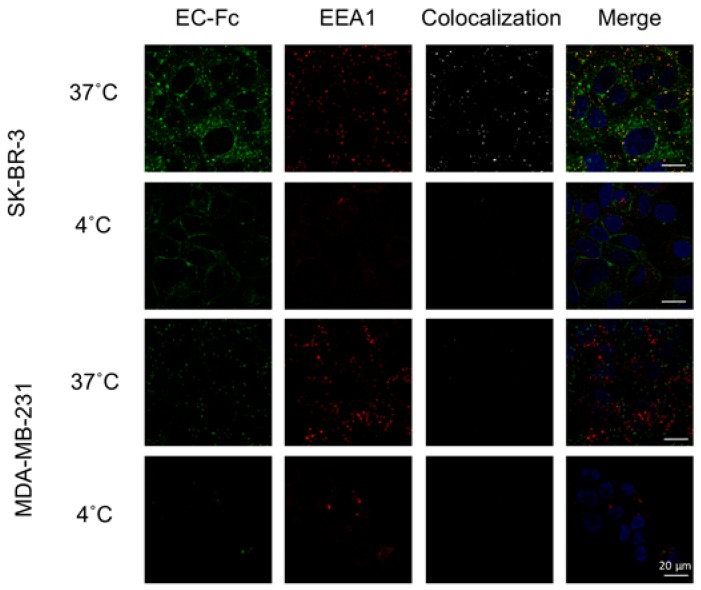
HER2 specific internalization of DTX-ECL. The potential of internalization of DTX-ECL into SK-BR-3 cells and MDA-MB-231 cells was evaluated under confocal microscopy. The cells were treated with DTX-ECL at either 4 °C or 37 °C for an hour. The localizations of EC-Fc (green) and EEA1 (red) were observed. Overlapping regions of EC-Fc and EEA1 in each treatment show in the right middle panels. All bars indicate 20 μm.

The HER2 specific internalization of DTX-ECL in HER2 overexpression cancer cells was further assessed using immunostaining assay under confocal microscopic observation. The localization of EC-Fc on DTX-ECL was evaluated in both SK-BR-3 cells and MDA-MB-231 cells after an hour exposure (Figure 5). EC-Fc dramatically internalized into SK-BR-3 under the condition at 37 °C but not at 4 °C. The EC-Fc colocalized with the early endosome marker, EEA1, indicating DTX-ECL was internalized via an endocytic pathway. Further, EC-Fc was not significantly detected in MDA-MB-231 when treated with DTX-ECL at both 37 °C and 4 °C. Collectively, DTX-ECL was considered to specifically target HER2 overexpressing cancer cells and internalize via HER2 mediated endocytic pathway.

## 3. Discussion

Liposomal taxanes are expected as promising drug formulations for cancer therapy. In this study, we have developed a method to encapsulate taxanes at feasible EE and LE into liposomes by suspending lipid film containing drugs with CEP. The resultant DTX-L was further conjugated with EC1 peptide to exert HER2 specific affinity on the surface to establish drug delivery system targeting HER2.

Incorporation of a surfactant such as Tween 80 into liposomes has been successfully demonstrated to generate liposomal taxanes [21,22,23,24]. Although surfactants cause side effects [37], the surfactant encapsulated into liposomes may reduce them because of the effects of liposomal drug delivery. In fact, our previous study showed that the CEP encapsulating liposomes alleviated acute toxicity of naked CEP in mice [13]. CEP thus should be a good candidate for the solvent of the inner hydrophilic core of liposomes encapsulating taxanes. To compare CEP and 3 volume% Tween 80, MLVs formulations were observed after suspension of lipid film, which was composed of HSPC, Chol, mPEG-DSPE, and either PTX or DTX at the molar ratio 60:40:5:10 or 60:40:5:20, respectively (Figure S1). While 3 volume% Tween 80 enhanced the formation of spindle-like morphology, presumably aggregates of lipid and taxanes, CEP generated clear MLVs. This different observation should be resulting from the solubility of taxanes in CEP superior to that in 3% Tween 80. In this context, suspension the lipid film with CEP appears to successfully provide the best-optimized formulation of liposomes encapsulating effective amounts of taxanes with over 90% of EE (Figure 1 and Figure 2). The LEs of the all liposomal taxanes were higher than that of gPTX-L prepared by solubility gradient method. Since our previous gPTX-L were intravenously injected at 75 mg/kg at single injection in mice, the liposomal taxanes in this study may be applicable with high dose of administration. The particle size of all the liposomes encapsulating taxanes ranged between 100 and 200 nm (Table S1), which is suitable for EPR effects [3]. These formulations of liposomal taxane were stable at least for a month at 4 °C without significant leakage of drug and change in particle size (Figure 3). 

Nanoparticle-based drugs including PTX encapsulated micelles was recently announced the disappointing results in the clinical trials [2]. Although liposomal doxorubicin alleviated the side effects such as cardiac toxicity, overall survival was not improved when compared with naked doxorubicin in metastatic breast cancer [38]. Possible reasons for these results might be traced to the restricted drug release from nanoparticles and the interaction between nanoparticles and stromal cells within the tumor microenvironment. In order to improve antitumor activity, functional liposomes including antibody-coupled liposomes targeting cancer have been investigated. Cancer-selective antibody and peptide should allow liposomes to selectively accumulate in the cancer tissues followed by internalization and/or membrane fusion to targeted cells to release the encapsulated drugs. We prepared liposomes targeting HER2 by coupling EC1 peptides on the surface of DTX-L to evaluate the potential of functional liposomal drug delivery system. While modification of EC-Fc on DTX-L caused leakage of the drug, the EE (69.3%) and the LE (21.3%) were still considered high efficiencies. The resultant DTX-ECL sustained good conditions including particle size, zeta potential, and morphology (Table 1 and Figure 2). DTX-ECL exhibited lower IC_50_ and higher uptake amounts of the drug than those by DTX-L in the HER2 positive cancer cells but not in the HER2 negative cells (Figure 4). Further, the analysis by confocal microscopy revealed EC-Fc on DTX-ECL was specifically internalized into HER2 overexpressing cancer cells and colocalized with EEA1, suggesting that DTX-ECL internalized into the cells via HER2 mediated pathway of endocytosis (Figure 5). When compared the cellular uptake of DTX between the treatments with naked DTX and DTX-ECL, naked DTX showed more uptake than DTX-ECL. However, the cytotoxicity of naked DTX was less than that of DTX-ECL in HER2 positive cancer cell lines. While naked DTX was passively penetrated into the cells, DTX encapsulated in DTX-ECL was internalized into the cells via HER2-mediated endocytosis pathway. The endocytotic internalization of DTX-ECL might help locally deliver the drug in an effective concentration to exert cytotoxicity more efficiently than naked DTX. Thus, DTX-L was successfully developed to drug delivery system targeting HER2 by coupling EC-Fc peptides on the surface of liposomes without significant impairment of the liposomal formulation. 

We demonstrated the successful generation of liposomal taxanes with high LE of the drugs as well as high EE using CEP in this study. Importantly, our PTX-L and DTX-L are composed of clinically available materials and the lipid composition is already applied in doxorubicin-encapsulating PEGylated liposomes, known as Doxil [14]. Further, the encapsulation method allowed DTX-L to develop targeted drug delivery. Although further studies are required to determine the pharmacokinetics and the antitumor activity of the liposomal taxanes in vivo, the liposomes prepared by the suspension in CEP should be a promising formulation for drug delivery of taxanes. 

## 4. Materials and Methods

### 4.1. Materials

Hydrogenated soybean phosphatidylcholine (HSPC), 1,2-distearoyl-*sn*-glycerol-3-phosphoethanolamine-*N*-[methoxy (polyethylene glycol)-2000] (mPEG–DSPE), and 1,2-distearoyl-*sn*-glycerol-3-phosphoethanolamine-*N*-[maleimide (polyethylene glycol)-2000] (Mal–PEG–DSPE) were obtained from NOF (Tokyo, Japan). Cholesterol (Chol) was purchased from Kanto Chemical (Tokyo, Japan). Thiazolyl blue tetrazolium bromide (MTT), RPMI 1640 medium, Dulbecco Modified Eagle Medium (DMEM), and polyethoxylated castor oil (Cremophor EL) were obtained from Sigma-Aldrich (St Louis, MO, USA). PTX and DTX were purchased from Tokyo Chemical Industry (Tokyo, Japan) and from Indena Japan (Tokyo, Japan), respectively. 7-glucosyloxyacetylpaclitaxel (gPTX) was synthesized as previously described [39]. 

### 4.2. Preparation of Liposomes Encapsulating Taxanes

DTX-, PTX-, or gPTX-encapsulating liposomes (DTX-L, PTX-L, or gPTX-L, respectively) were prepared by thin-film hydration method. HSPC, Chol, and mPEG-DSPE (60:40:5 molar ratio) with taxanes (DTX, PTX, or gPTX) were dissolved in an organic solvent (chloroform: methanol = 9:1 volume ratio). The solvent was evaporated using rotary evaporator at 45 °C. The resulting lipid film was left overnight under vacuum to completely dry. The resulting lipid film was suspended in CEP (Cremophor EL: ethanol: PBS, pH 7.4 (10 mM PO_4_^3−^, 137 mM NaCl, and 2.7 mM KCl) = 20:15:65 volume ratio) at 60 °C, which formed multi-lamellar vesicles (MLVs). MLVs were then sonicated three times by the Sonicator 3000 (Misonix, Farmingdale, NY, USA) equipped with 3.2 mm micro tip at 60 °C for 5 min or 10 min to DTX-L and gPTX-L or PTX-L to form small lamellar vesicles (SLVs). The outer solvent of the liposomes was replaced to PBS by ultrafiltration using a 100K-membrane filter (Merck Millipore, Billerica, MA, USA). 

### 4.3. Evaluation of the Leakage of Drugs from Liposomes

The taxanes-encapsulating liposomes were kept at 4 °C in PBS. After 2 weeks and 4 weeks, the liposomes were washed with PBS by ultrafiltration using the 100K-membrane filter to remove leakage drugs from the liposomes. The drugs retained in liposomes were determined using a reverse-phase high-performance liquid chromatography (HPLC) described below. The retention rate was calculated as a ratio of drug retained in liposomes to initial drug in liposomes.

### 4.4. Preparation of EC1 Peptide Fused to Human IgG Fc Domain (EC-Fc)

EC-Fc was prepared as previously described [40,41]. EC-Fc was produced from Chinese hamster ovary (CHO) cells that stably transfected with pBO853 DNA carrying a coding sequence for EC-Fc. The CHO cells were cultured using a bioreactor, miniPERM (SARSTEDT, Nümbrecht, Germany). Twenty million of the cells were suspended in 50 mL of CHO-S-SFM II (Life Technologies, Grand Island, NY, USA) supplied with 100 μg/mL Hygromycin B (Nakarai Tesque, Kyoto, Japan) and were transferred into production module. The production module was connected to nutrient module contained 450 mL of CHO-S-SFM II supplied with 100 μg/mL Hygromycin B. The bioreactor was rotated for 10 days at 37 °C in 5% CO_2_. The medium in production module was then collected and centrifuged at 150× *g* for 5 min at 4 °C to remove the cells. The supernatant was re-centrifuged at 10,000× *g* for 5 min at 4 °C. The supernatant was then passed thorough 0.20 μm filter to completely remove cell debris. EC-Fc was then purified as follows. The supernatant was passed through a 0.5 mL of Protein A Sepharose (GE Healthcare, Uppsala, Sweden) equilibrated with PBS. After washing the column with PBS, EC-Fc was eluted using 0.1 M sodium phosphate buffer at pH 2.6. Five hundred μL of each fraction was readily neutralized with 10 μL of 2 M sodium phosphate buffer, pH 8.0. The fraction containing EC-Fc was detected by western blotting using horseradish peroxidase (HRP) conjugated anti-human IgG antibody and the concentration was determined using a BCA assay kit (Pierce Biotechnology, Rockford, IL, USA). 

### 4.5. Preparation of DTX-L Conjugated with EC1 Peptide (DTX-ECL)

EC-Fc was conjugated on the surface of DTX-L using maleimide and thiol reaction to prepare DTX-ECL [13]. DTX contained lipid film composed of HSPC, Chol, mPEG-DSPE, and Mal-PEG-DSPE (60:40:4.75:0.25 molar ratio) were prepared. The lipid film was formed liposomes as described above. EC-Fc was reacted with 2-iminothiolane (Sigma-Aldrich) at the molar ratio of 1:50 in 25 mM HEPES, pH 8.0 containing 140 mM NaCl for an hour at room temperature in the dark to induce thiol groups. The EC-Fc was passed through a PD-10 column (GE Healthcare, Uppsala, Sweden) to replace the solvent with PBS. Finally, Maleimide groups on liposomes and thiol groups on EC-Fc were then reacted for 2 h at room temperature. Unbounded EC-Fc was removed by ultrafiltration using a 300K-membrane filter (Merck Millipore Ltd., Billerica, MA, USA).

### 4.6. Evaluation of Encapsulation Efficiency (EE) and Loading Efficiency (LE)

Encapsulation efficiency (EE) was calculated as the ratio of the amount of taxanes encapsulated into liposomes to the initial amount of the drug. Loading efficiency (LE) was calculated as the molar ratio of the drug encapsulated in liposomes to the total of phospholipids. The amount of encapsulated drug was evaluated by the revers-phase HPLC equipped a solvent pump (L2130, Hitachi, Aichi, Japan), a UV detector (L2400, Hitachi) and a hydrophobic C_18_ column (150 mm × 4.6 mm, 4 μm, GL Sciences Inc., Tokyo, Japan) The moving phase, 70% (*v*/*v*) methanol, was flowed at 1 mL/min. Ten μL of each sample was injected and the drug was detected 227 nm for PTX and gPTX and at 229 nm for DTX.

### 4.7. Characterization of Liposomes

The particle size and zeta potential of liposomes were determined by dynamic light scattering and electrophoretic light scattering with ELS-8000 (Otsuka Electronics, Osaka, Japan). The liposomal formulation of DTX-L, DTX-ECL, PTX-L, and gPTX-L were observed using transmission electron microscopy (TEM) as previously described [13]. The TEM study was conducted by Hanaichi Ultra-Structure Research Institute (Aichi, Japan).

### 4.8. Cell Culture

The human colon cancer cell line HT-29 and the human breast cancer cell line SK-BR-3 and MDA-MB-231 were purchased from ATCC (Rockville, MD, USA). HT-29 cells and SK-BR-3 cells were cultured in RPMI 1640 medium supplemented with 10% fetal bovine serum (FBS, PAA Laboratories, Pasching, Austria). MDA-MB-231 cells were cultured in DMEM with 10% FBS. Cells were maintained at 37 °C in an atmosphere of 5% CO_2_.

### 4.9. In Vitro Evaluation of Cellular Uptake of the Drugs

Cellular uptake of the drugs in the different formulations was evaluated as previously described [26]. Cells were seeded at 2.0 × 10^7^ cells/well in 12-well plate and were incubated for overnight. The drug was then added at the concentration of 10 μM. After incubation for 3 h at 37 °C, the cells were washed with ice-cold PBS for three times and were collected by trypsinization. After centrifugation at 1000× *g* for 5 min, the supernatant was removed and the cell pellet was suspended in 100 μL of 10% (*w*/*v*) sodium dodecyl sulfate. The proteins in the solution were precipitated by adding 100 μL of acetonitrile. After centrifugation at 12,000× *g* for 5 min, the concentration of the drug in the supernatant was determined by reverse-phase HPLC as described above.

### 4.10. Cytotoxicity Assay

Cytotoxicity of the different formulations of taxanes was evaluated by an MTT assay [33]. Cells were seeded in 96-well plate at 5000 cells/well and incubated for overnight. The different concentrations of the drugs were added to each well. After 3-h incubation, the medium was changed to fresh one without the drug. After 72-h from when addition of the drugs, the cell viability was evaluated by the MTT assay as described previously [33]. The concentration at which cell growth was inhibited by 50% (IC_50_) was estimated from survival curve. Each experiment was performed four times to calculate standard deviation.

### 4.11. Confocal Microscopic Observation

The internalization of EC-Fc on DTX-ECL was observed under a confocal microscope (FV-1000, Olympus, Tokyo, Japan). SK-BR-3 cells or MDA-MB-231 cells were seeded on a gelatin-coated glass coverslip in 24-well plate at 1.8 × 10^5^ cells/well and incubated for overnight. The cells were then incubated in serum-free medium for an hour at either 4 °C or 37 °C. DTX-ECL at the lipid concentration of 500 mM in serum-free medium was then added. After incubation for an hour at indicated temperature, the cells were fixed using 4% paraformaldehyde for 15 min at room temperature. After the cells were permeabilized using 0.1% Triton-X in PBS for 5 min, 4% BSA in PBS was added to block unspecific binding of the antibodies for an hour at room temperature. Rabbit anti-EEA1 antibody (Cell Signaling Technology, Danvers, MA, USA) were added and incubated for an hour at room temperature. Cy3-conjugated anti-human-IgG (Fc-specific) antibody (Sigma-Aldrich) and Alexa488-conjugated anti-Rabbit-IgG antibody (Thermo Fisher Scientific, Waltham, MA, USA) was then added at room temperature in the dark for 30 min. After mounted the coverslip on the slide glass with antifade mountant with DAPI (Vector Laboratories, Burlingame, CA, USA), the cells were observed under the confocal microscopy with proper lasers and filters.

### 4.12. Statistical Analysis

The results are presented as the mean ± standard deviation (SD). Statistical significance was evaluated using two-tailed *t*-test where necessary. *P* < 0.05 was considered statistically significant.

## 5. Conclusions

The liposomal formulation using CEP as a solvent of the inner hydrophilic core of liposomes has great potential to encapsulate large amounts of taxanes including DTX, paclitaxel and glycosylated paclitaxel. The PEGylated liposomes encapsulating taxanes exhibited good conditions for EPR effects and were stable in the storage condition. HER2 specific binding peptide could confer the HER2 targeting potential to the liposomes encapsulating DTX. The DTX-ECL exhibited significantly enhanced cytotoxic activity on HER2 overexpression cancer cells in vitro. Therefore, the liposomal formulation prepared with CEP is concluded as a promising candidate of liposomal drug delivery system for taxanes.

## Figures and Tables

**Table 1:** Characteristics of DTX-L and DTX-ECL.

Drug Formulation	EE (%)	LE (%)	Particle Size (nm)	Zeta Potential (mV)	PDI
DTX-L	97.3 ± 2.0	29.9 ± 0.6	148.1 ± 16.1	−4.65 ± 1.25	0.130 ± 0.030
DTX-ECL	69.3 ± 9.7	21.3 ± 3.0	186.7 ± 5.4	−7.10 ± 2.80	0.154 ± 0.013

All data are depicted as mean ± S.D. where *N* = 4.

## Supplementary Materials

The following are available online at http://www.mdpi.com/2079-4991/7/10/290/s1, Figure S1. MLV formulations after suspension of lipid film containing taxanes with CEP or 3 volume% Tween 80, Figure S2. Cytotoxicity of liposomal taxanes after 72 h drug exposure, Table S1. Characteristics of liposomes encapsulating taxanes.
