# Supplementary material for: Practical Liposomal Formulation for Taxanes with Polyethoxylated Castor Oil and Ethanol with Complete Encapsulation Efficiency and High Loading Efficiency
Source: Nanomaterials (Basel). 2017 Sep 23;7(10):290. doi: 10.3390/nano7100290 (PMC5666455; doi:10.3390/nano7100290)
Supplement: Supplementary file 1 [file nanomaterials-07-00290-s001.pdf]

Supplementary Nanomaterials: Practical Liposomal Formulation for Taxanes with Polyethoxylated Castor Oil and Ethanol with Complete Encapsulation Efficiency and High Loading Efficiency

Tsukasa Shigehiro [1,2], Junko Masuda [1], Shoki Saito [1], Apriliana C. Khayrani [1], Kazumasa Jinno [1], Akimasa Seno [1], Arun Vaidyanath [1], Akifumi Mizutani [1], Tomonari Kasai [1], Hiroshi Murakami [1], Ayano Satoh [1], Tetsuya Ito [3], Hiroki Hamada [4], Yuhki Seno [5], Tadakatsu Mandai [5] and Masaharu Seno [1,*].

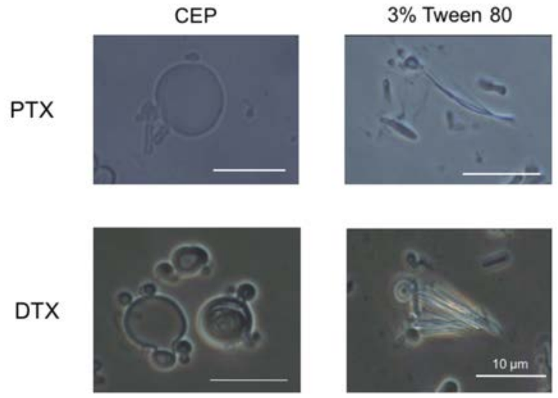

Figure S1. MLV formulations after suspension of lipid film containing taxanes with CEP or 3 volume% Tween 80. Lipid-film composed of HSPC, Chol, mPEG-DSPE, and either PTX or DTX (60:40:5:10 or 60:40:5:20 molar ratio, respectively) was suspended in either CEP or 3% Tween 80. Once the lipid film was suspended in indicated solvent, MLVs were observed under an inverted microscope (IX81, Olympus, Tokyo, Japan) with an objective lens of 100x magnification. The bars indicate 10 µm.

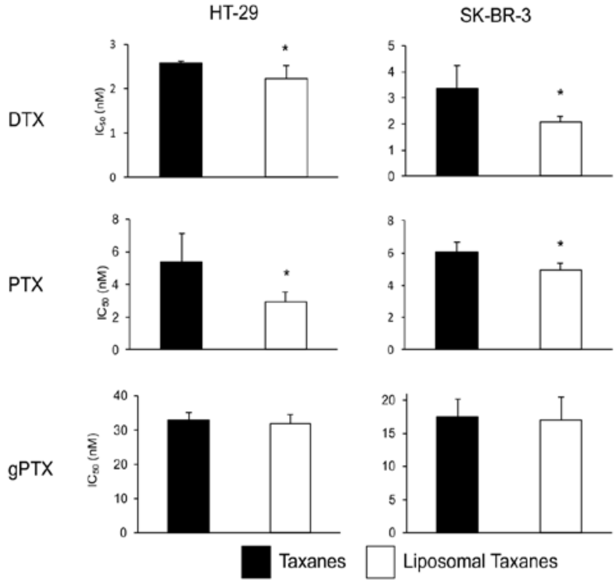

Figure S2. Cytotoxicity of liposomal taxanes after 72 h drug exposure. The IC_{50}s of the liposomal taxanes after 72 h drug exposure were determined from survival curve assessed by the MTT assay. * $P<0.05$; $N = 5$.

Table S1. Characteristics of liposomes encapsulating taxanes.

Drug formulation	Drug ratio (mol%)	Particle size (nm)	Zeta potential (mV)	PDI
DTX-L	10	163.4 ± 18.2	-7.66 ± 4.62	0.131 ± 0.018
	20	148.1 ± 16.1	-4.65 ± 1.25	0.130 ± 0.030
	30	185.7 ± 14.2	-5.22 ± 3.05	0.115 ± 0.021
PTX-L	5	127.2 ± 27.1	-3.91 ± 2.96	0.197 ± 0.019
	10	128.4 ± 22.6	-4.21 ± 2.22	0.165 ± 0.013
	20	142.3 ± 12.0	-5.77 ± 4.00	0.198 ± 0.122
gPTX-L	10	174.5 ± 16.5	-4.00 ± 1.46	0.162 ± 0.020
	20	171.3 ± 19.7	-4.18 ± 2.84	0.174 ± 0.022
	30	161.3 ± 25.5	-3.21 ± 1.42	0.189 ± 0.023

All data are depicted as mean ± S.D. where $N = 4$.
